# The complete chloroplast genome sequence of *Fargesia qinlingensis* (Poaceae): an endemic to China

**DOI:** 10.1080/23802359.2018.1473739

**Published:** 2018-05-18

**Authors:** Ya Fu Zhou, Yu Chao Wang, Xi Lu Ni, Shao Li Mao

**Affiliations:** a Xi’an Botanical Garden of Shaanxi Province/Institute of Botany of Shaanxi Province, Xi’an, China;; b Shaanxi Engineering Research Centre for Conservation and Utilization of Botanical Resources, Xi’an, China;; c Ningxia Forestry Institute, Yinchuan, China

**Keywords:** *Fargesia qinlingensis*, chloroplast genome, endemic species

## Abstract

The complete chloroplast genome of *Fargesia qinlingensis* (Poaceae) has been reconstructed from the whole-genome Illumina sequencing data. The complete chloroplast genome sequence of *F*. *qinlingensis* obtained in this study was 139,640 bp in length, with a large single copy (LSC) region of 83,220 bp, a small single copy (SSC) region of 12,826 bp, separated by two inverted repeat (IR) regions of 21,797 bp each. The GC content was 38.9%, and in the LSC, SSC, and IR regions were 37.0%, 33.2%, and 44.2%, respectively. A total of 130 genes were annotated, including 83 protein-coding genes (PCGs), 39 transfer RNA (tRNA) genes, and eight ribosomal RNA (rRNA) genes. The result of the phylogenetic analysis showed that *F*. *qinlingensis* was more closely related to the species of *F*. *denudata*.

*Fargesia qinlingensis* (Poaceae), a Chinese endemic species, is mainly found in a special ecological zone from the elevation of 1600–3000 m in Mt. Qinling, Shaanxi, China (Liang 2000). It is also acting as a crucial food for the Giant panda (Liu and Jin 2008), and the growth status and the productivity of *F*. *qinlingensis* have a significant effect on the present living situation of the Giant panda (Li et al. 2016). The complete chloroplast genome of *F*. *qinlingensis* (Poaceae) has been reconstructed using Illumina pair-end sequencing data. The specific goals of the present study were to present the complete chloroplast genome sequences of *F*. *qinlingensis*, and provide new data for studying phylogenetic relationship with other plants and conservation of the endemic species in Poaceae. The annotated chloroplast genome sequence of this species has been submitted to GenBank with the accession number MH117939.

Leaf materials from *F*. *qinlingensis* (Poaceae) were obtained from Mt. Qinling, Shaanxi, China (33°44′40.01″N, 107°49’15.85’’E). The voucher specimens of *F*. *qinlingensis* were deposited in Institute of Botany of Shaanxi Province. The complete chloroplast genome of *F*. *qinlingensis* was sequenced using Illumina HiSeq 2500 (Illumina, ‎San Diego, CA), yielding 10,978,383 reads which were then quality trimmed with CLC Genomics Workbench 8 (CLC Bio, Aarhus, Denmark). The programs of MITObim v1.8 (GitHub, San Francisco, CA) (Hahn et al. 2013) and Geneious v 10.1.2 (Biomatters Ltd., Auckland, New Zealand) were respectively employed for assembled and annotation of chloroplast genome, and *Fargesia spathacea* (GenBank: JX513417) was used as the initial reference.

The chloroplast genome sequence of *F*. *qinlingensis* was 139,640 bp in length and composed of large single copy (LSC) region of 83,220 bp, small single copy (SSC) region of 12,826 bp and two inverted repeat (IR) copies 21,797 bp. The order and orientation of the gene arrangement pattern of *F*. *qinlingensis* was identical with that of *F. spathacea*. The GC content was 38.9%, and in the LSC, SSC, and IR regions were 37.0%, 33.2%, and 44.2%, respectively. A total of 130 genes were annotated, including 83 protein-coding genes (PCGs), 39 transfer RNA (tRNA) genes, and eight ribosomal RNA (rRNA) genes. Eighteen genes (*rps19, trnH-GUG, rpl2, rpl23, trnI-CAU, trnL-CAA, ndhB, rps7, trnV-GAC, rrn16, trnI-GAU, trnA-UGC, rrn23, rrn4.5, rrn5, trnR-ACG, trnN-GUU, rps15*) were duplicated in the IR regions. For the trans-spliced rps12 gene, a 114bp 5′ end exon in the LSC region and a 3′ end with two exons (267 bp) were located in the IR region. Eight tRNA genes (*trnK-UUU, trnG-UCC, trnL-UAA, trnV-UAC, trnI-GAU, trnA-UGU, trnA-UGC*) and 10 PCGs (*rps16, atpF, ydf3, rps12, petB, petD, rpl16, rpl2, ndhB, ndhA*) contained one or two introns. The result showed that *F*. *qinlingensis* was more closely related to the species of *F*. *denudata* (Figure 1).

**Figure 1.**
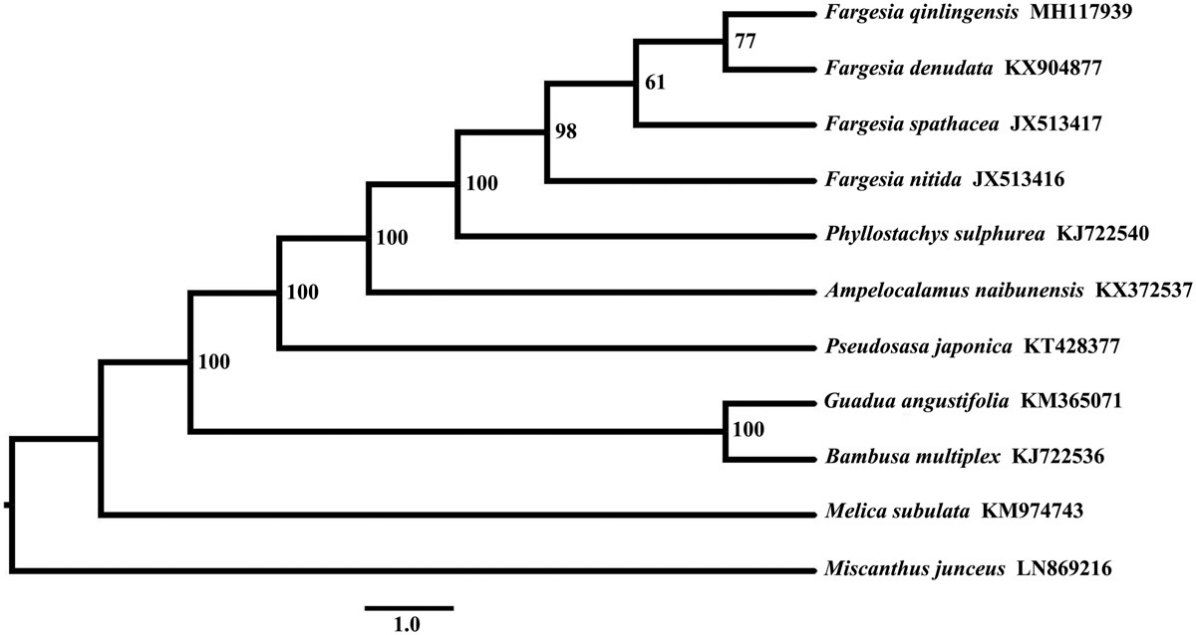
For phylogenetic tree construction, the chloroplast genome of *F. qinlingensis* was aligned with other eight species of Bambusoidea and two outgroups (*Melica subulata* and *Miscanthus junceus*).
